# The Response Regulator BfmR Is a Potential Drug Target for *Acinetobacter baumannii*

**DOI:** 10.1128/mSphere.00082-16

**Published:** 2016-05-11

**Authors:** Thomas A. Russo, Akshay Manohar, Janet M. Beanan, Ruth Olson, Ulrike MacDonald, Jessica Graham, Timothy C. Umland

**Affiliations:** a Veterans Administration Western New York Healthcare System, Buffalo, New York, USA; b Department of Medicine, University at Buffalo, State University of New York, Buffalo, New York, USA; c Department of Microbiology and Immunology, University at Buffalo, State University of New York, Buffalo, New York, USA; d Department of Structural Biology, University at Buffalo, State University of New York, Buffalo, New York, USA; e The Witebsky Center for Microbial Pathogenesis, University at Buffalo, State University of New York, Buffalo, New York, USA; f Hauptman Woodward Medical Research Institute, Buffalo, New York, USA; University of Rochester

**Keywords:** *Acinetobacter baumannii*, Gram-negative bacilli, antibiotic target, bacterial drug target, drug discovery, essential genes, multidrug resistant

## Abstract

Identification and validation is the first phase of target-based antimicrobial development. BfmR (RstA), a response regulator in a two-component signal transduction system (TCS) in *Acinetobacter baumannii*, is an intriguing potential antimicrobial target. A unique characteristic of BfmR is that its inhibition would have the dual benefit of significantly decreasing *in vivo* survival and increasing sensitivity to selected antimicrobials. Studies on the clinically relevant strain AB307-0294 have shown BfmR to be essential *in vivo*. Here, we demonstrate that this phenotype in strains AB307-0294 and AB908 is mediated, in part, by enabling growth in human ascites fluid and serum. Further, BfmR conferred resistance to complement-mediated bactericidal activity that was independent of capsular polysaccharide. Importantly, BfmR also increased resistance to the clinically important antimicrobials meropenem and colistin. BfmR was highly conserved among *A. baumannii* strains. The crystal structure of the receiver domain of BfmR was determined, lending insight into putative ligand binding sites. This enabled an *in silico* ligand binding analysis and a blind docking strategy to assess use as a potential druggable target. Predicted binding hot spots exist at the homodimer interface and the phosphorylation site. These data support pursuing the next step in the development process, which includes determining the degree of inhibition needed to impact growth/survival and the development a BfmR activity assay amenable to high-throughput screening for the identification of inhibitors. Such agents would represent a new class of antimicrobials active against *A. baumannii* which could be active against other Gram-negative bacilli that possess a TCS with shared homology.

**IMPORTANCE** Increasing antibiotic resistance in bacteria, particularly Gram-negative bacilli, has significantly affected the ability of physicians to treat infections, with resultant increased morbidity, mortality, and health care costs. In fact, some strains of bacteria are resistant to all available antibiotics, such as *Acinetobacter baumannii*, which is the focus of this report. Therefore, the development of new antibiotics active against these resistant strains is urgently needed. In this study, BfmR is further validated as an intriguing target for a novel class of antibiotics. Successful inactivation of BfmR would confer the multiple benefits of a decreased ability of *A. baumannii* to survive in human body fluids, increased sensitivity to complement-mediated bactericidal activity and, importantly, increased sensitivity to other antibiotics. Structural studies support the potential for this “druggable” target, as they identify the potential for small-molecule binding at functionally relevant sites. Next-phase high-throughput screening studies utilizing BfmR are warranted.

## Abstract (precis)

Increasing antibiotic resistance in bacteria, particularly Gram-negative bacilli, has significantly affected the ability of physicians to treat infections, with resultant increased morbidity, mortality, and health care costs. In fact, some strains of bacteria are resistant to all available antibiotics, such as *Acinetobacter baumannii*, which is the focus of this report. Therefore, the development of new antibiotics active against these resistant strains is urgently needed. In this study, BfmR is further validated as an intriguing target for a novel class of antibiotics. Successful inactivation of BfmR would confer the multiple benefits of a decreased ability of *A. baumannii* to survive in human body fluids, increased sensitivity to complement-mediated bactericidal activity and, importantly, increased sensitivity to other antibiotics. Structural studies support the potential for this “druggable” target, as they identify the potential for small-molecule binding at functionally relevant sites. Next-phase high-throughput screening studies utilizing BfmR are warranted.

## INTRODUCTION

The incidence of infections due to multidrug-resistant, extensively resistant, and pan-drug-resistant (MDR, XDR, and PDR) Gram-negative bacilli (GNB) is increasing, with *Acinetobacter baumannii* one of the most problematic species (1–8). Treatment of infections due to these strains has become challenging, with resultant increased morbidity, mortality, and health care costs (9–11). The promise of a postantibiotic era is on the cusp of being fulfilled for *A. baumannii* (1–8, 12, 13), and a true PDR strain has been reported (14). This reality has been appreciated for a number of years, with the 2004 report by the Infectious Diseases Society of America, *Bad Bugs, No Drugs: As Antibiotic R&D Stagnates, a Public Health Crisis Brews*, being one of several attempts to increase exposure and enable solutions (15–20). However, development of new antimicrobials is a long, arduous, and expensive process. As a result, many major pharmaceutical companies left the antimicrobial research and development (R&D) business (19, 21–23). The burden of early-stage development has fallen in part to academia and to “start-up” companies. The term ESKAPE pathogens was coined to advertise the major culprits, with *Klebsiella pneumoniae*, *A. baumannii*, *Pseudomonas aeruginosa*, and *Enterobacter* spp. being the initial GNB implicated. *A. baumannii*, with its propensity of being XDR and PDR, has been termed the “poster child” for this ever-increasing threat to health care. The need to identify new antimicrobials active against *A. baumannii* is more pressing than ever. Therefore, as a first step, our group identified *A. baumannii* essential genes *in vivo* (i.e., those required for bacterial growth and survival in a host) which represent potential novel drug targets (24), with the long-term goal to develop a new class of antimicrobials. In support of such efforts, new regulatory paths may make the development of focused treatments for XDR pathogens, such as *A. baumannii*, commercially viable (25).

One of the potential targets identified as essential *in vivo* was BfmR (24). BfmR is the response regulator of a two-component signal transduction system (TCS) which propagates the signal from its corresponding sensor histidine kinase BfmS and acts as a transcriptional regulator (26). The environmental stimuli that the BfmR/BfmS (also referred to RstAB) system responds to are unknown, other than a direct or indirect effect of sub-MICs of chloramphenicol in the strain ATCC 17978 (27). Such systems have been considered potential drug targets (28), and the histidine kinase QseC has been successfully targeted by using the small organic molecule LED209 (29). Although LED209 inhibited virulence *in vivo*, it did not inhibit pathogen growth. Since inactivation of BfmR resulted in loss of viability and was essential *in vivo* (24) and important for *in vivo* pathogenicity in a murine pneumonia model (30), its successful targeting may result in even greater efficacy than that observed for antimicrobials targeting QseC. Phenotypes conferred by BfmR include increased resistance to complement-mediated bactericidal activity, biofilm formation, and scaffolding of the outer membrane protein OmpA, as well as increased resistance to selected antimicrobials (e.g., carbapenems) (27, 31, 32). In this report, we extend our initial biological observations on BfmR and add structural insights focused on its receiver domain (residues 1 to 130; BfmR^1‑130^) that further support this protein as a potential drug target in *A. baumannii*.

## RESULTS

### Disruption of BfmR synthesis affects growth/survival in human ascites fluid and serum *ex vivo*, but not in rich laboratory medium.

**FIG 1:**
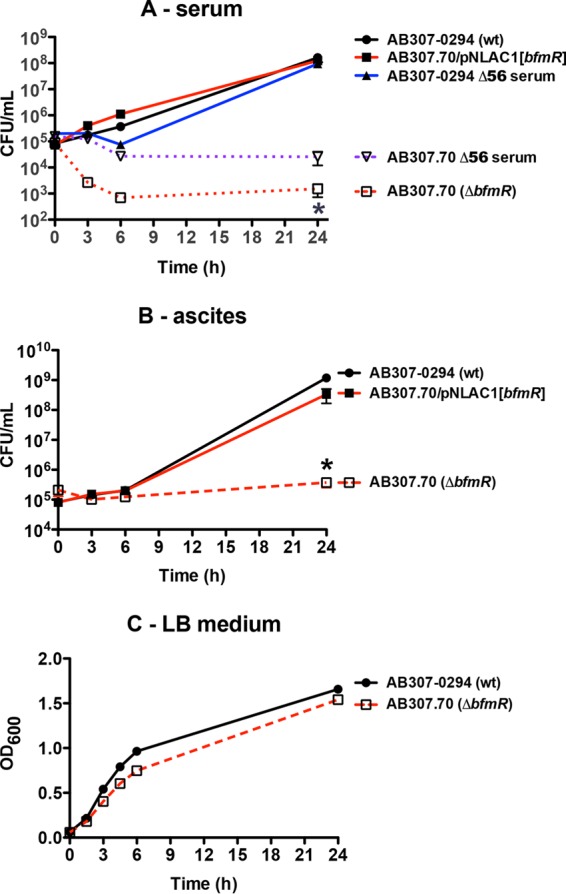
Comparison of the growth/survival of AB307-0294 (wt) and AB307.70 (Δ*bfmR*) cells in 90% human serum, 100% human ascites fluid, and rich laboratory medium. (A) Growth/survival in serum was assessed by measurement of CFU at 0, 3, 6, and 24 h. AB307-0294 and AB307.70 were also grown in serum that was heated to 56°C for 30 min, which inactivated complement-mediated bactericidal activity (Δ56 serum). AB307.70/pNLAC1[*bfmR*], a BfmR-complemented derivative of AB307.70, was also tested to confirm that the observed phenotype was BfmR mediated. Data are means ± SEM (*n =* 6). (B) Growth/survival in ascites fluid was assessed by measurement of CFU at 0, 3, 6, and 24 h. Data are means ± SEM (*n =* 6 to 8). (C) Growth in LB medium, as measured by the OD_600_ of four replicate cultures per strain. Data are means ± SEM (*n* = 4). *, *P* < 0.05/2 (two-tailed unpaired *t* test for AB307-0294 compared to AB307.70 in untreated serum and ascites fluid).

A critical criterion for a potential drug target is essentiality. We have previously published data that demonstrated BfmR as being essential *in vivo* in a rat infection model (24). To extend this observation, we performed quantitative growth/survival studies in human serum and ascites fluid samples. AB307.70 (Δ*bfmR*) underwent a 2-log complement-mediated decrease in growth/survival over 24 h in serum (Fig. 1A). This loss in growth/survival was restored to wild-type (wt) levels when AB307.70 was complemented in *trans* (AB307.70/pNLAC1[*bfmR*]). Surprisingly, when AB307.70 was grown in serum in which complement activity had been inactivated (Δ56°C), growth was not restored to wild-type levels, but instead an approximate 1-log loss in growth/survival was observed (Fig. 1A). Importantly, the decrease in growth/survival of AB307.70 was significantly greater in untreated serum than in serum in which complement was inactivated. Likewise, AB307.70 underwent a greater-than-3-log decrease in growth/survival when grown in human ascites fluid, and this loss in growth/survival was again complemented in *trans* (AB307.70/pNLAC1[*bfmR*]) (Fig. 1B). These data further support an essential role for BfmR for optimal growth/survival of AB307-0294 in these clinically relevant environments. In contrast and, importantly, AB307.70 is capable of growth in rich laboratory medium, with a slight decrease in log-phase growth but a similar plateau for cell density as with AB307-0294, thereby excluding a generalized growth defect (Fig. 1C). These data demonstrate that BfmR mediates (directly or indirectly) resistance to complement-mediated bactericidal activity. These data also demonstrate that BrmR regulates bacterial factors needed for optimal growth, presumably via nutrient acquisition, since decreased growth of AB307.70 was observed in serum compared to growth of its wild-type parent when complement was inactivated.

### Disruption of BfmR synthesis has no effect on capsule production.

**FIG 2:**
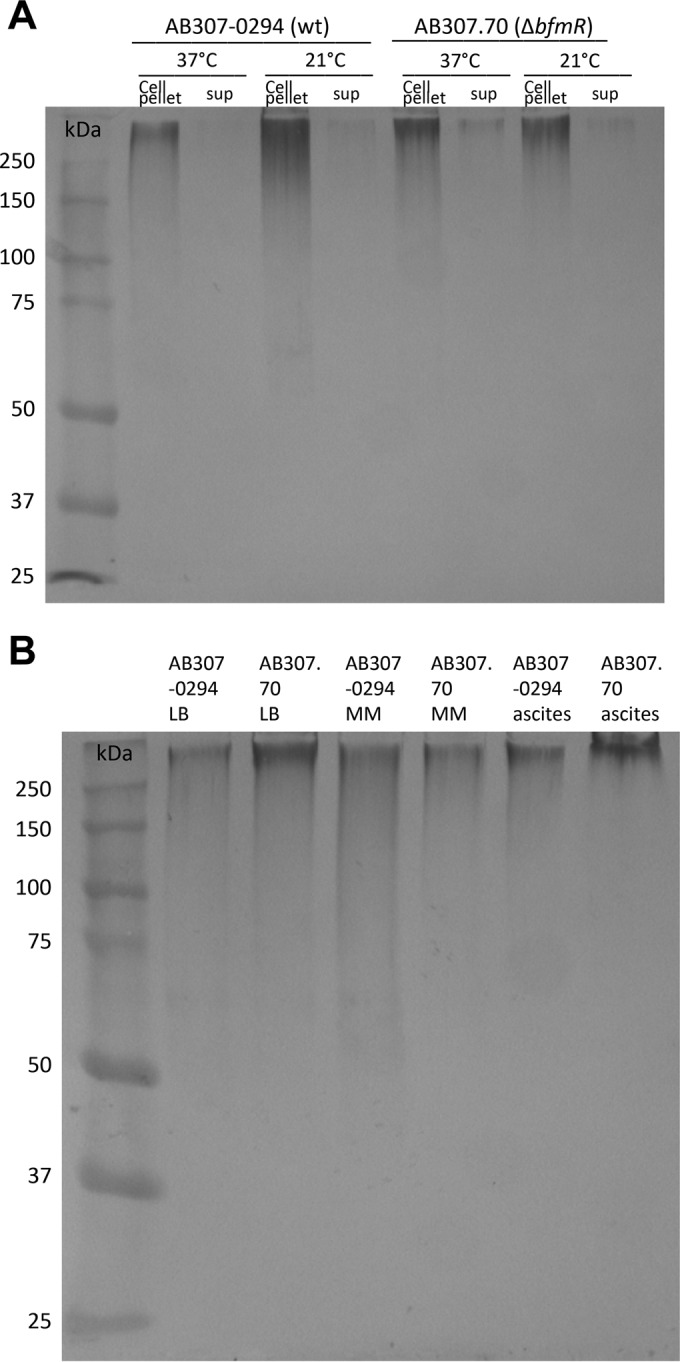
Comparison of capsular polysaccharide production by AB307-0294 (wt) and AB307.70 (Δ*bfmR*). Capsule production was assessed by Western blotting. The monoclonal antibody 13D6, which has been shown to recognize the K1 capsule produced by AB307-0294, was used to detect capsule. (A) Cell-associated capsule (cell pellet) and capsule in culture supernatant (sup) was assessed after AB307-0294 and AB307.70 were grown in rich Luria-Bertani laboratory medium at 37°C and 21°C. (B) Cell-associated capsule was assessed after AB307-0294 and AB307.70 were grown in rich LB laboratory medium, minimal medium (MM), or human ascites fluid at 37°C.

Previously published work by our group demonstrated that in *A. baumannii* the capsular polysaccharide was a major factor contributing to resistance of complement-mediated bactericidal activity (33). Therefore, as a first step in examining the mechanism by which BfmR confers serum resistance, the production of the K1 capsular polysaccharide in AB307-0294 (wt) and AB307.70 (Δ*bfmR*) was assessed. Both strains were grown in LB medium at either 21°C (room temperature) or 37°C, and cell lysates or supernatant fractions underwent Western blot analysis using the monoclonal antibody (MAb) 13D6, which has been shown to recognize the K1 capsule present on AB307-0294 (33). Surprisingly, capsule was produced in roughly equal amounts by both AB307-0294 and AB307.70 (Fig. 2A). Further, the majority of capsular polysaccharide was cell associated. Next, Western blot analysis was performed to determine if growth at 37°C in minimal medium and human ascites fluid (in which complement was inactivated to enable the growth of AB307.70) affected the production of the K1 capsular polysaccharide in AB307-0294 and AB307.70 (Fig. 2B). These data clearly demonstrated that the loss of BfmR does not decrease capsule production. Taken together, these data do not support BfmR as a positive regulator of capsule production. Further, the mechanism by which BfmR confers resistance to complement-mediated bactericidal activity appears to be independent of capsule production.

### BfmR increases resistance to the antimicrobials meropenem and colistin.

**FIG 3:**
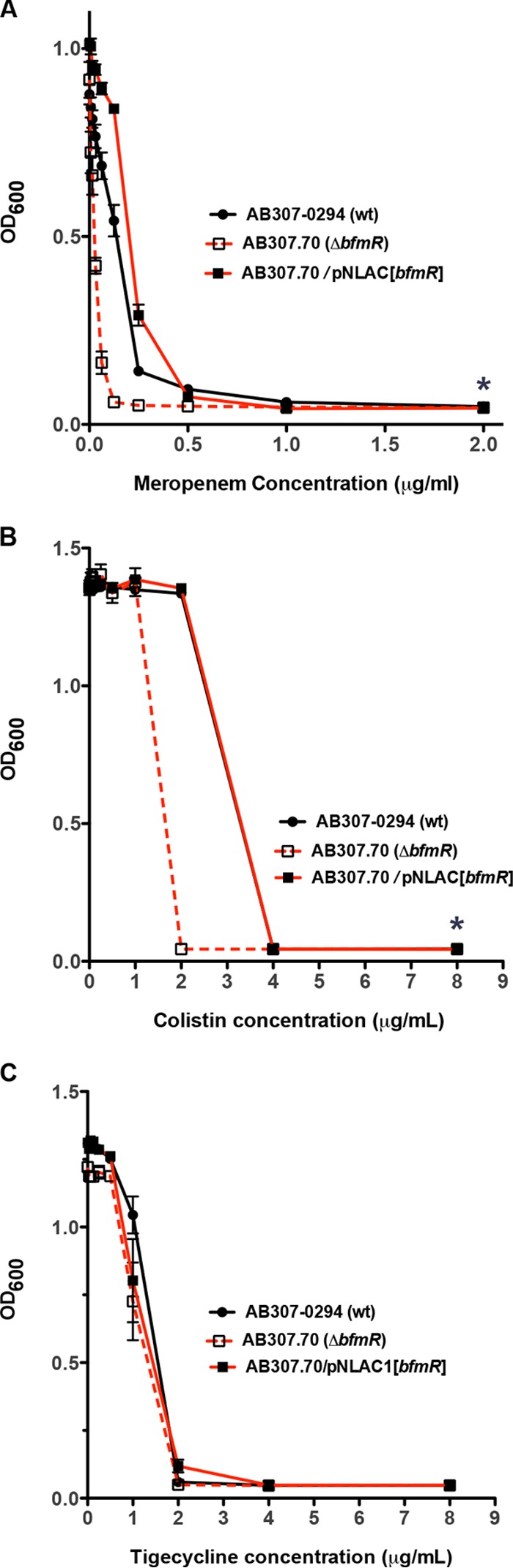
Comparison of the growth/survival of AB307-0294 (wt), AB307.70 (Δ*bfmR*), and AB307.70/pNLAC[*bfmR*] after exposure to various concentrations of meropenem (A), colistin (B), or tigecycline (C). Bacterial growth/survival was assessed by measuring the OD_600_ after overnight growth in Müeller-Hinton broth in the presence or absence of various concentrations of meropenem (0 µg/ml, 0.0078 µg/ml, 0.0156 µg/ml, 0.0312 µg/ml, 0.0625 µg/ml, 0.125 µg/ml, 0.25 µg/ml, 0.5 µg/ml, 1.0 µg/ml, and 2.0 µg/ml) or colistin/tigecycline (0 µg/ml, 0.0312 µg/ml, 0.0625 µg/ml, 0.125 µg/ml, 0.25 µg/ml, 0.5 µg/ml, 1.0 µg/ml, 2.0 µg/ml, 4.0 µg/ml, and 8.0 µg/ml). Data are means ± standard errors of the means (*n =* 9 to 15 for each strain at each concentration of antimicrobial). *, *P* < 0.05/2 (two-tailed unpaired *t* test for AB307-0294 compared to AB307.70).

Previous studies had suggested that BfmR caused increased resistance to colistin (polymyxin E), rifampin, erythromycin, and imipenem (27, 31). However, these studies used the *A. baumannii* type strain ATCC 17978, which is of unclear clinical significance (34). Therefore, we assessed the sensitivities of AB307-0294 (wt), AB307.70 (Δ*bfmR*), and AB307.70/pNLAC1[*bfmR*] to the carbapenem antimicrobials meropenem, colistin, and tigecycline. BfmR did not affect the activity of tigecycline (Fig. 3C). In contrast and consistent with prior studies (27, 31), BfmR significantly increased resistance to meropenem and colistin (Fig. 3A and B). The MIC for meropenem decreased from 1.0 µg/ml to 0.125 µg/ml, and for colistin it decreased from 2.0 µg/ml to 1.0 µg/ml with the loss of BfmR. These data demonstrate that BfmR mediates an increased level of resistance to two important classes of antimicrobials.

### Disruption of BfmR synthesis in a second *A. baumannii* clinical isolate (AB908Δ*bfmR*) does not affect capsule production and decreases growth/survival in human ascites fluid and serum *ex vivo*.

**FIG 4:**
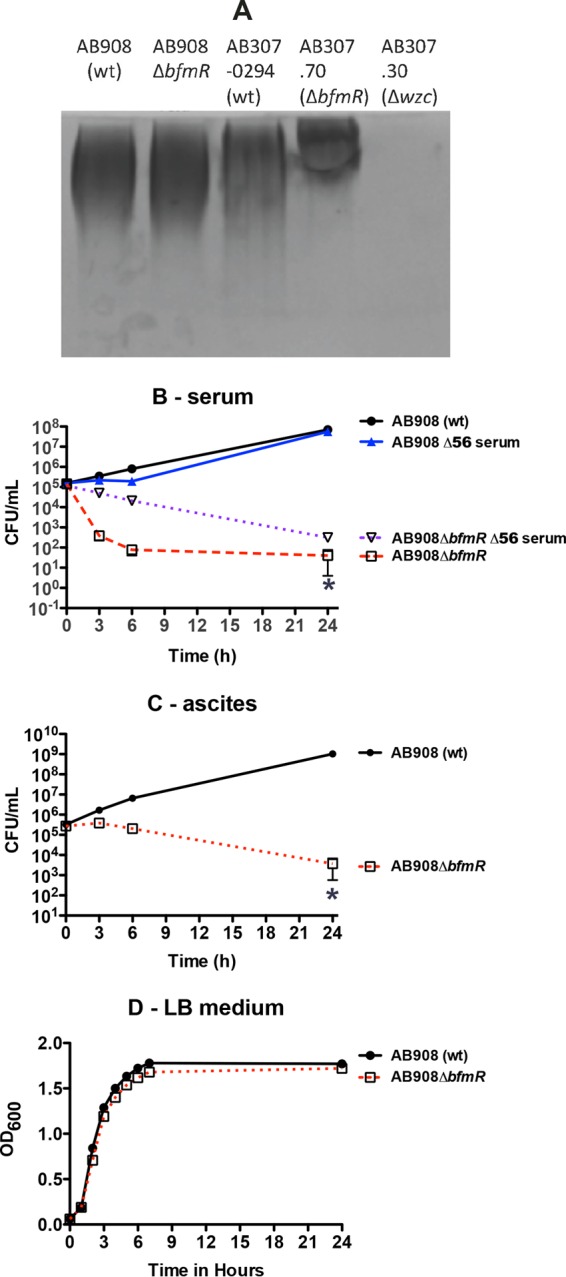
Comparison of capsular polysaccharide production and growth/survival of AB908 (wt) and AB908Δ*bfmR* in 90% human serum, 100% human ascites fluid, or rich laboratory medium. (A) Capsule production was assessed by alcian blue staining. Cell-associated capsule (cell pellet) was assessed after AB908 (wt) and AB908Δ*bfmR* were grown in rich Luria-Bertani laboratory medium at 37°C. AB307-0294 (wt, capsule positive), AB307.70 (Δ*bfmR*, capsule positive), and AB307.30 (Δ*wzc*, capsule negative) served as positive and negative controls. (B) Growth/survival in serum was assessed by measurement of CFU at 0, 3, 6, and 24 h. AB908 (wt) and AB908Δ*bfmR* were also grown in serum that was heated to 56°C for 30 min, which inactivated complement-mediated bactericidal activity (Δ56 serum). Data are means ± SEM (*n* = 5 to 7). (C) Growth/survival in ascites fluid was assessed by measurement of CFU at 0, 3, 6, and 24 h. Data are means ± SEM (*n* = 4). (D) Growth in LB medium, as measured by the OD_600_. Data are means ± SEM (*n* = 4). *, *P* < 0.05/2 (two-tailed unpaired *t* test for AB908 [wt] compared to AB908Δ*bfmR* in untreated serum and ascites fluid).

To confirm that the phenotypic effect of BfmR was not unique to AB307-0294, a site-directed disruption of *bfmR* was created in the contemporary clinical isolate AB908, resulting in the construct AB908Δ*bfmR*. As was observed for AB307-0294, disruption of *bfmR* did not affect capsule production (Fig. 4A), resulted in decreased growth/survival in human ascites fluid and serum (Fig. 4B and C), and demonstrated similar growth in rich laboratory medium compared to its wild-type parent (Fig. 4D). Likewise, as observed for AB307.70, when AB908Δ*bfmR* was grown in serum in which complement activity was inactivated (Δ56°C), growth was not restored to wild-type levels (Fig. 4B). These data support that the phenotypic effects of the disruption of BfmR synthesis were similar in 2 clinically relevant strains of *A. baumannii*.

### BfmR is not homologous to human proteins and is conserved among isolates of *A. baumannii*.

A desirable trait of a potential antimicrobial drug target is a lack of homology to human proteins in order to enable target specificity. An amino acid sequence similarity search against the human proteome revealed a lack of any human proteins homologous to BfmR. This was an expected result, as TCSs are not known to be present in humans and other mammals (35, 36).

A second desirable trait of an antimicrobial target is overall protein conservation, and perhaps more importantly, conservation of the targeted drug binding site across bacterial species and strains of interest. BfmR consists of 238 amino acids, has a predicted molecular mass of 27.1 kDa, and has a predicted pI of 4.98. A BLASTp search of all nonredundant *A. baumannii* protein sequences (14 July 2015 data set) identified 908 proteins that were ≥99% identical (majority, 100%; over ≥99% coverage) to AB307-0294 BfmR. This analysis was complemented using the OMA orthologue database (September 2014 release) (37), which employs only high-quality complete genomic sequences. BfmR was 100% prevalent with fully identical sequences in the eight *A. baumannii* strains annotated in the OMA (*O*rthologous *MA*trix) database (AB307-0294, ATCC 17978, 0057, AYE, ACICU, SDF, 1656-2, and TCDC-AB0715) plus the two *A. baumannii* strains sequenced by our group in addition to AB307-0294: AB853 (blood isolate from Iraq) and AB979 (environmental isolate from Iraq) (unpublished data). Additionally, two other *Acinetobacter* species present within OMA, *A. baylyi* ADP1 (a nonpathogenic soil bacterium) and *A. calcoaceticus* PHEA-2 (an industrial wastewater isolate) both contained a BfmR orthologue with fully identical amino acid sequences. These data support that BfmR is conserved across *A. baumannii* strains.

### BfmR^1-130^ crystal structure.

The ability to determine a crystal structure is an important tool for characterizing ligand-protein interactions during downstream drug development. *A. baumannii* BfmR is predicted to belong to the OmpR/PhoB response regulator family based on sequence analysis, and it contains a receiver domain (BfmR^1-130^) and a DNA binding effector domain (BfmR^131-238^). BfmR^1-130^ was selected for structural studies, as conformational changes within the receiver domain dictate DNA binding activity of the effector domain. BfmR^1-130^ was a promiscuous crystallizer, with multiple hits observed in the automated 1536 condition crystallization screen. A selection of chemically diverse crystallization conditions were evaluated for optimization, but in each case the resulting BfmR^1-130^ crystals were relatively small. Evaluation of X-ray diffraction by crystals grown under a condition that was inherently cryoprotective demonstrated that the rod-like crystals of 0.15 to 0.20 mm length but only ~0.03 mm in diameter, with a hexagonal cross-section, produced data usable for the structure determination (Table 1).

**TABLE 1:** X-ray data collection and refinement statistics

Characteristic	Value^a^
PDB code	5E3J
Data collection	
Space group	P 6_5_
Unit cell parameters (Å)	*a* = *b* = 52.0, *c* = 197.9
Resolution range (Å)	50.00–2.10 (2.18–2.10)
Completeness (%)	99.3 (94.3)
Total no. of reflections	189,638
No. of unique reflections	17,580 (1,668)
Multiplicity	10.8 (6.5)
*R*_merge_	0.086 (0.401)
〈 I/σ(I)〉	20.5 (4.4)
Wilson *B* factor (Å^2^)	24.1
Refinement	
Resolution range (Å)	29.73–2.10 (2.18–2.10)
No. of reflections, working set	16,282 (1,737)
No. of reflections, test set	1,229 (132)
*R*_cryst_/*R*_free_	0.1651 (0.2200)/0.1938 (0.2420)
No. of non-H atoms	
Protein/water	1,929/181
Model geometry (root-mean-square deviations from ideal)	
Bonds (Å)/angles (°)	0.004/0.83
Average *B* factors (Å^2^)	
Protein/water	31.3/40.5
Ramachandran plot (%)^b^	
Favored/allowed/outlier	100/0/0
MolProbity clashscore^b^	1.26
Rotamer outliers (%)^b^	0.94

a Values in parentheses are for the highest resolution shell.

b As calculated by MolProbity; MolProbity clashscore corresponds to 100th percentile (i.e., best) among structures of comparable resolution. $R_{\mathrm{merge}}=\frac{\Sigma_{\mathrm{hkl}}\;\Sigma_{j}\;\left|I_{\text{hkl},\;j}-〈I_{\text{hkl}}〉\right|}{\Sigma_{\mathrm{hkl}}\;\Sigma_{j}\;I_{\mathrm{hkl},j}}$ $R_{\mathrm{cryst}}\;\mathrm{or}\;R_{\mathrm{free}}=\frac{\Sigma_{\mathrm{hkl}}\left|F_{\mathrm{hkl}}^{\mathrm{obs}}-F_{\mathrm{hkl}}^{\mathrm{calc}}\right|}{\Sigma_{\mathrm{hkl}}\;F_{\mathrm{hkl}}^{\mathrm{obs}}}$ R_cryst_ and *R*_free_ were calculated using working and test data sets, respectively.

The BfmR^1-130^ crystal structure was readily determined by molecular replacement, using the PhoP receiver domain from *Bacillus subtilis* (PDB 1MVO; protein sequence 50% identical and 73% similar over 119 residues) as the search model. The BfmR^1-130^ crystal structure asymmetric unit contained two protomers, associated as a dimer by a noncrystallographic 2-fold rotation (root mean square [RMS] deviation for the C-α atom superposition of the two protomers is 0.18 Å). The space group was determined to be P6_5_, with symmetry-related reflections scaling much poorer in the higher-symmetry P6_5_22 space group (R_merge_, 8.6% versus 35.2%, respectively, to 2.10-Å resolution). The final refined model possessed a defining electron density for Lys6-Thr125 for both protomers, plus 181 modeled solvent molecules. The fold of an individual BfmR^1-130^ protomer is highly similar to the α_5_-β_5_ doubly wound motif observed for other OmpR/PhoB family receiver domains, as expected (38).

### BfmR^1-130^ homodimerization.

The OmpR/PhoB family of response regulators is activated by autophosphorylation of a conserved aspartate residue in the receiver domain (Asp58 in BfmR). Phosphorylation induces an equilibrium shift favoring the active conformation, which includes formation of a 2-fold symmetrical homodimer involving the α4-β5-α5 face of the receiver domain. This homodimeric conformation facilitates the effector domain to bind DNA and modulate transcription (39, 40). A small molecule that interrupts this activation in BfmR would be a potential drug-lead candidate. In the BfmR^1-130^ crystal structure described here, Asp58 was not subjected to phosphorylation or the presence of a phosphorylation mimic (e.g., BeF^3−^). Moreover, the divalent cation (Mg^2+^ or Mn^2+^) required for autophosphorylation was not observed in the electron density in the cleft neighboring Asp58. Thus, BfmR^1-130^ is expected to be in an inactive state (i.e., a monomer or a nonproductive homodimer complex) within this crystal structure.

**FIG 5:**
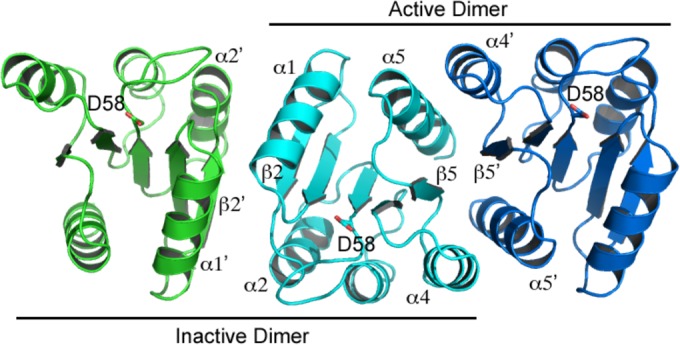
The BfmR^1-130^ crystal structure exhibits two distinct homodimer interfaces. The OmpR/PhoP family canonical active homodimer (α4-β5-α5 interface; cyan and blue chains) was observed despite the unphosphorylated state of Asp58. A unique and presumably inactive homodimer (α1-β2-α2 interface; cyan and green chains) was also observed.

**FIG 6:**
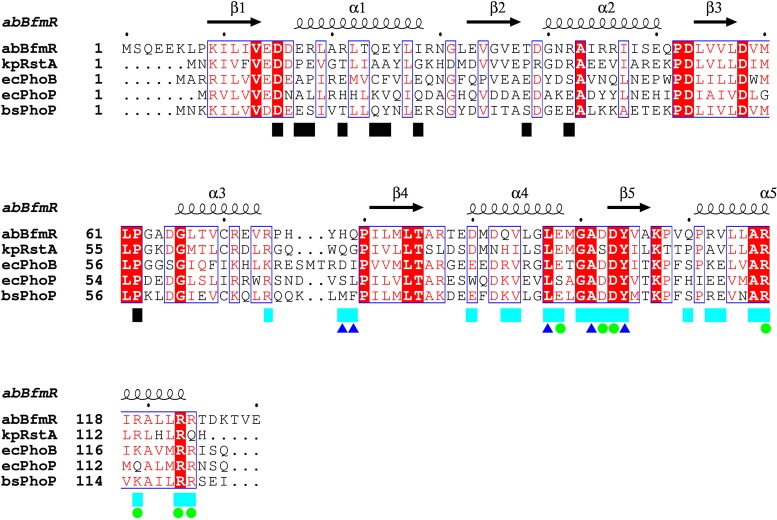
Alignment of the BfmR^1-130^ sequence against representative OmpR/PhoP family receiver domains. Fully conserved residues are contained in red filled boxes, and highly conserved residues are in blue outlined boxes. Secondary structure elements are based on the *A. baumannii* BfmR^1-130^ crystal structure. Black and cyan boxes denote residues participating in the α1-β2-α2 and α4-β5-α5 homodimer interfaces, respectively; blue triangles denote residues with interface interactions limited to main chain atoms, and green circles denote residues participating in interprotomer salt bridges in *A. baumannii* BfmR^1-130^. Sequence abbreviations: abBfmR, *A. baumannii* BrmR; kpRstA, *K. pneumoniae* RstA; ecPhoB, *E. coli* PhoB; ecPhoP, *E. coli* PhoP; bsPhoP, *B. subtilis* PhoP.

The BfmR^1-130^ crystal structure exhibited a noncrystallographic 2-fold symmetric homodimer in the asymmetric unit, formed by the α1-β2-α2 face of each protomer (Fig. 5). This α1-β2-α2 interface buried a total of 906 Å^2^ (6.7% of the total surface area). A search of the PDB revealed that this specific homodimer interface has not been previously observed for response regulator receiver domains. Moreover, the BfmR residues that mediate this interface are poorly to modestly conserved in other OmpR/PhoB family receiver domains (Fig. 6). A second BfmR^1-130^ noncrystallographic 2-fold symmetric homodimer was also present, unexpectedly exhibiting the α4-β5-α5 interface typical of OmpR/PhoB family receiver domains in the active phosphorylated conformation. This homodimer was comprised of a protomer A and a protomer B from two neighboring asymmetric units. This interface buried a total of 2,051 Å^2^ (or 15.3% of the total surface area), similar to that observed in crystal structures of receiver domains activated using BeF^3−^ (41, 42). Furthermore, the amino acid composition of the BfmR^1-130^ α4-β5-α5 interface was highly similar to that present in other OmpR/PhoB family receiver domains (Fig. 6). For example, comparison of the BfmR^1-130^ structure with the analogous interface in BeF_3_^−^-activated *Escherichia coli* PhoB (PDB 1ZES) revealed that of the 21 interface residues per protomer, 12 (57%) are identical and 5 (24%) are similar in PhoB. By comparison, the overall residue identity between these two receiver domains is 39%. The four residue positions that are significantly different at this interface are BfmR His78, Gln79, Gln93, and Val113 versus PhoB Asp76, Ile77, Arg91, and Glu111, respectively. The BfmR His78-Gln79/PhoB Asp76-Ile77 residues participate at their respective dimer interfaces through formation of hydrogen bonds between their main chain carbonyl oxygens to the guanidinium group of BfmR Arg124′/PhoB Arg122′, respectively, where the prime symbol indicates a residue belongs to the partner protomer within the dimer. In PhoB, a salt bridge is formed across the dimer interface by Arg91 and Glu111′. The positionally equivalent residues in BfmR, Gln93 and Val113′, are incapable of participating in an analogous interfacial salt bridge. However, to partially compensate for the loss of this stabilizing interaction in BfmR, Gln93 participates in a hydrogen bond with the guanidinium group of Arg117′ across the interface.

**FIG 7:**
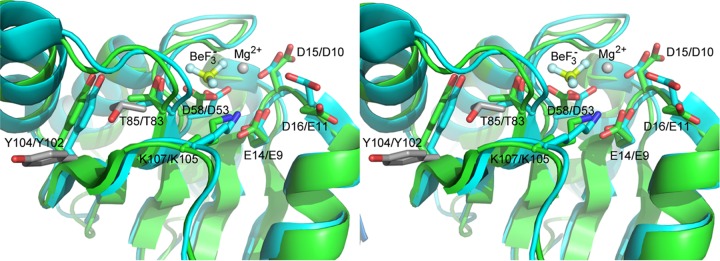
Stereoview of cartoon representations of *A. baumannii* BfmR^1-130^ (cyan) superimposed upon the activated *E. coli* PhoB receiver domain (PDB 1ZES; green). The conserved active site and conformational switch residues are displayed as sticks. Labels are in the following format: BfmR residue/PhoB residue. Residues present only in the *E. coli* PhoB structure are BeF3^−^, which mimics phosphorylation of D53, and the active site Mg^2+^ ion. The BfmR^1-130^ conformational switch residues T85 and Y104 are in the canonical active conformation, similar to those (T83 and Y102) in activated PhoB. The inactive side chain conformations of these two PhoB residues are displayed in gray.

The BfmR^1-130^ α4-β5-α5 dimer interface is also three-dimensionally similar to the typical OmpR/PhoB family receiver domain active homodimer structure. The RMS deviations for superimposed C-α atoms from BfmR^1-130^ and *E. coli* PhoB (PDB 1ZES) individual protomers range between 0.96 and 1.08 Å, versus 1.12 Å for the superimposed α4-β5-α5 homodimers. In addition to homodimerization, the canonical activation mechanism involves a characteristic shift of a serine or threonine switch residue toward the phosphorylated aspartate, such that the side chain hydroxyl group forms a hydrogen bond to the phosphate (42). An aromatic residue (tyrosine or phenylalanine) on the β5 strand undergoes a rotamer change to shift its bulky side chain away from the α4-β5-α5 dimerization interface and into the volume previously occupied by the Ser/Thr side chain. The BfmR^1-130^ crystal structure exhibits the active conformation of these two residues (Thr85 and Tyr104), further suggesting that this structure represents the active form of the BfmR receiver domain, despite the absence of phosphorylation or a phosphorylation mimic (Fig. 7).

### Prediction of small-molecule binding hot spots.

**FIG 8:**
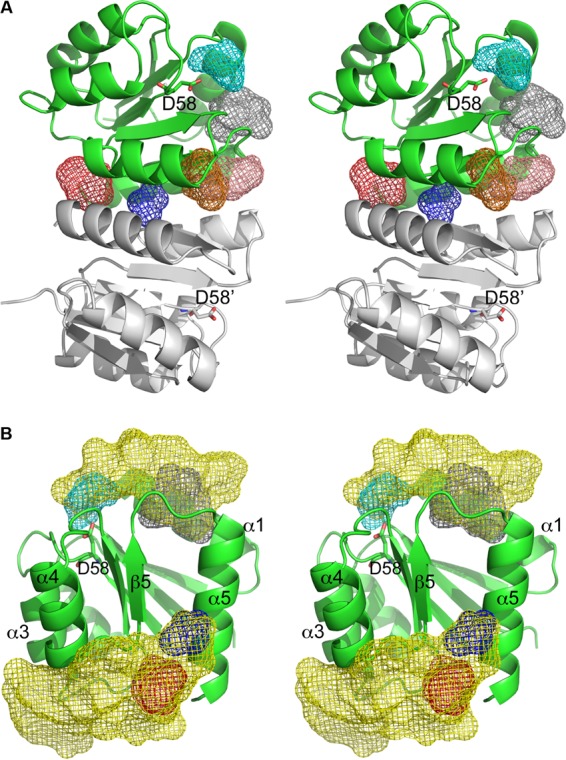
Stereoviews of BfmR^1-130^ structural features as potential antimicrobial targets. (A) Results of the FTMAP prediction of ligand-binding hot spots at functionally important locations, displayed as mesh-enclosed volumes, on the surface of a BfmR^1-130^ protomer (represented in green). The binding hot spots are color-coded according to the predicted importance (red, strong; salmon, strong; orange, medium; cyan, medium; blue, weak), which correlated to the number of bound small molecules predicted to cluster at that location. A sixth predicted binding hot spot neighboring the phosphorylation site is represented by gray mesh. The second protomer (gray ribbon cartoon) illustrates the homodimer organization, but it was excluded from the binding hot spot calculations. For clarity, only predicted hot spots near the functionally important α4-β5-α5 face and Asp58 are displayed. (B) Stereoview of preferential docking locations of drug-like molecules at functionally important regions of the BrmR^1-130^ protomer. The yellow mesh encompasses volumes where drug-like molecule preferentially docked on the BfmR^1-130^ protomer surface. These preferential docking locations encompass several of the binding hot spots identified by FTMAP (red and blue mesh on the α4-β5-α5 face and cyan and gray mesh at and near the phosphorylation site).

Computational predictions (via the FTMap server) of small-molecule binding hot spots (i.e., surface regions with major contributions to ligand binding free energy) on BfmR^1-130^ were performed to estimate the likelihood that small-molecule inhibitors acting specifically through the BfmR receiver domain can be identified (43). This analysis predicted 11 binding hot spots on the BfmR^1-130^ protomer surface, which could be classified as very strong (1 predicted hot spot), strong (3 predicted hot spots), medium (3 predicted hot spots), or weak (4 predicted hot spots). It was hypothesized that a BfmR inhibitor would likely bind at the α4-β5-α5 face, thereby preventing productive formation of the active BfmR homodimer, or it would bind near Asp58 to inhibit phosphorylation. Six binding hot spots were identified at these locations (Fig. 8A). A medium binding hot spot was present in the cleft near Asp58, occupying the volume where phosphorylation would occur. A single weak hot spot was predicted on the middle of the α4-β5-α5 face, in a shallow cleft between strand β5 and helix α5, and one medium and two strong hot spots were predicted on the periphery of this dimerization face. A sixth predicted binding hot spot was located neighboring the phosphorylation site, between helix α1 and the N terminus of helix α5.

### Screening of drug and drug-like virtual libraries.

The binding hot spot predictions were based upon docking of chemically simple probes composed of 2 to 8 nonhydrogen atoms. The potential for BfmR to bind more elaborate drug-like small molecules was further evaluated through a blind docking strategy. This *in silico* analysis was performed by docking virtual libraries representative of FDA-approved, NCI diversity 3 and LOPAC1280 compounds over the entire BfmR receptor domain protomer surface to predict preferential binding sites. Two clusters of high-scoring docked molecules were present about a subset of the FTMap-predicted binding hot spots at functionally important locations, indicative of locations likely to be suitable for binding drug-like molecules (Fig. 8B). Specifically, one cluster was at the dimer interface occupying both the binding hot spot at the cleft formed by the N terminus of strand β5 and the C terminus of α5 (red mesh in Fig. 8B) and the hot spot at the center of the α4-β5-α5 face (blue mesh in Fig. 8B). Binding interactions for this cluster also extended under the C-terminal ends of helices α3 and α4. A second cluster occupied a binding hot spot in the cleft between helix α1 and the N terminus of helix α5 (gray mesh in Fig. 8B). Several molecules in this docking cluster extended to also occupy the binding hot spot located in the phosphorylation site (cyan mesh in Fig. 8B).

## DISCUSSION

Findings from this report further support BfmR as a potential drug target of *A. baumannii*. An important and highly appealing characteristic of BfmR is that its inhibition would have the dual benefit of significantly decreasing *in vivo* survival as well as increasing the sensitivity of *A. baumannii* to other antimicrobials.

A biological property that supports this concept is the previous demonstration that BfmR is essential for survival *in vivo* (24). Here, we have demonstrated a mechanism responsible for this phenotype. BfmR enhances growth/survival in human ascites fluid and serum, which was mediated, at least in part, by conferring resistance to complement-mediated bactericidal activity (Fig. 1A and B and 4B and C). A recent report that studied *A. baumannii* strain ATCC 17978 also demonstrated that BfmR increased resistance to complement-mediated bactericidal activity and that this phenotype was mediated by decreased capsule production (27). However, the mechanism by which BfmR mediates resistance to complement-mediated bactericidal activity in AB307-0294 and AB908, the strains assessed in this study, appears to be different. Although our group previously demonstrated that capsular polysaccharide confers resistance to complement-mediated bactericidal activity (33), in AB307-0294 BfmR does not affect K1 capsule production (Fig. 2). An alternate explanation would be the production of a second, non-K1 capsular polysaccharide. However, surface polysaccharide was purified by extraction with pyridine acetate (44) from AB307-0294 (wt) and AB307::Tn*17* (both capsule positive) and mutant constructs with disruptions in either a K1 nucleotide-sugar biosynthetic gene (*gna*; AB307::Tn*69*) or a K1 capsule export gene (*wzc*; AB307.30) (both K1 capsule negative) (33, 45), resolved by SDS–8% polyacrylamide gel electrophoresis and subjected to alcian blue staining. A capsule was observed for AB307-0294 and AB307::Tn*17*, but not AB307::Tn*69* or AB307.30, thereby excluding a cryptic second capsule (data not shown). Therefore, the mechanism by which BfmR confers resistance to complement-mediated bactericidal activity is independent of capsule production. This difference is likely strain dependent. AB908 is a contemporary clinical isolate, and the model pathogen AB307-0294 is a blood isolate that was frozen from the primary clinical microbiology laboratory culture, thus maintaining its genetic integrity. It possesses a complete capsule, is resistant to 90% human serum, and is virulent in rat pneumonia and soft tissue infection models and a mouse systemic infection model (46). Disruption of the capsule transport gene *wzc* results in the loss of capsule but is not lethal (33), and capsule is primarily cell associated (this study). In contrast, the ATCC 17978 strain produces a thin capsule (27), is relatively sensitive to complement-mediated bactericidal activity (34), disruption of *wzc* appears to be a lethal mutation (27), significant amounts of capsule are detected in culture supernatant (27), and it is less virulent in both mouse and *Galleria mellonella* infection models (34, 47). Therefore, ATCC 17978 does not appear to be an optimal clinical representative of *A. baumannii*, making the translational implications of studies using this strain unclear.

Although the effect of BfmR on capsule production was discordant between AB307-0294 and ATCC 17978, its effect on conferring increased resistance to selected antimicrobials was similar (27, 31). Previous published studies demonstrated that BfmR increased resistance to colistin, imipenem, rifampin, and erythromycin, but not chloramphenicol, ceftazidime, amikacin, or ciprofloxacin (27, 31). These data are consistent with the concept that BfmR confers increased resistance to certain antimicrobials. Limitations of those studies were the use of ATCC 17978, which is not an optimal representative of *A. baumannii*, and the fact that erythromycin and chloramphenicol are not antimicrobials usually considered for the treatment of infections due to *A. baumannii*. In this report, we used a clinically relevant strain and antimicrobials. The mechanism that mediates this effect is unclear; however, a previous report demonstrated that BfmR affected the scaffolding of OmpA (31). It is tempting to speculate that alterations in outer membrane proteins could affect the sensitivity to certain antimicrobials. Regardless of the mechanism, these data add further support to BfmR as an antimicrobial target, since its inactivation would also have the additional beneficial effect of increasing its sensitivity to other antimicrobials.

Lastly, although we did not assess the effect of BfmR on biofilm formation, other investigators have demonstrated that it mediates increased biofilm production (32). Biofilms can directly contribute to infection (e.g., intravascular access or implanted devices). Therefore, targeting BfmR may have further benefit for these difficult-to-treat infections.

Understanding the structure of a potential drug target may provide valuable information for guiding drug development. For TCS response regulators, structure-function insights may identify possible ligand binding sights that will modulate formation of the active homodimer. BfmR exhibited several interesting structural features. The active homodimeric conformation of BfmR^1-130^ was observed despite not undergoing phosphorylation or phosphorylation mimicked by BeF_3_^−^ or other means. This unexpected active form has been observed previously for other OmpR/PhoB family receiver domains. It is thought that the high protein concentration present during crystallization may shift the equilibrium from monomer (or a nonproductive dimer) to the active homodimeric form (41, 42, 48, 49). Conversely, the crystallization conditions and resulting crystal lattice packing may favor the active BfmR homodimer.

The computational prediction of small-molecule binding hot spots on BfmR^1-130^ supports the possibility of identifying small-molecule inhibitors of BfmR activity that act by specific binding to the receiver domain. Binding hot spots on the BfmR^1-130^ protomer were predicted on the protomer face that participates in the active homodimer interface and at the phosphorylation site. It is possible that ligand binding at other hot spots outside these two regions may inhibit BfmR activity through an allosteric mechanism, but that is beyond the scope of this study, as is consideration of the possibility of inhibitor binding to the DNA-binding domain.

A predicted strong binding hot spot (red mesh in Fig. 8A) lies between the N terminus of strand β5 and the C terminus of α5, and the Arg124 side chain fits into this niche on the partner protomer of the homodimer. A small molecule bound in this niche would likely interrupt the Arg124 hydrogen bond to the His78′ carbonyl oxygen and the salt bridge to the Asp102′ carboxyl group. A second predicted strong hot spot (orange mesh in Fig. 8A) and the predicted weak hot spot (blue mesh in Fig. 8A) neighbor the Arg117 side chain, and a small molecule bound in either location would likely interfere with formation of the Arg117-Asp103′ salt bridge across the interface. Given the conserved nature of the OmpR/PhoB family receiver domain α4-β5-α5 face, it may be possible to develop a small molecule that inhibits activation of a broad range of response regulators. Interestingly, an *in silico* ligand-screening study for the *E. coli* PhoP receiver domain focused upon binding sites near PhoP residues Arg111 (analogous to BfmR Arg117) and Arg118 (analogous to BfmR Arg124), as these residues are key determinants of PhoP homodimerization and activation (50). Importantly, ligand binding hot spot prediction has been demonstrated to be less sensitive to protein conformational changes than the *in silico* prediction of a specific binding site for a specific ligand (43).

Blind docking results supported that several but not all predicted binding hot spots may be suitable for participating in binding interactions with drug-like molecules that will interfere with BfmR activation. However, this analysis also suggested that successful binding of drug-like molecules likely requires interactions with two binding hot spots or neighboring regions. For example, the phosphorylation site by itself was not a promising drug-like molecule binding site. Rather, docked drug-like molecules bound at the phosphorylation site also required binding interactions at the neighboring binding hot spot between helix α1 and the N terminus of helix α5. The phosphorylation site pocket is comprised primarily of charged and polar residues. A general feature of drug-protein complexes is the presence of hydrophobic in addition to charged or polar amino acids at the binding site (51), and so the phosphorylation site is not optimal for interacting with drug-like molecules but may participate in a larger binding site.

Computational methods predicted that BfmR is likely a druggable target. However, development of robust BfmR activity assays are required prior to further experimental exploration to identify and characterize hit or lead molecules for therapeutic development. Cell permeability and efflux pumps are major barriers in the identification of tractable Gram-negative antimicrobial lead molecules (52). Thus, an assay to evaluate efficacy of a compound with a BfmR-specific mechanism of action in the context of live *A. baumannii* is required. Furthermore, an assay to determine the threshold of BfmR inhibition necessary to result in therapeutically relevant antimicrobial activity is also needed, as a small-molecule drug may not completely inhibit the BfmRS signaling pathway *in vivo*. Previous efforts to identify small-molecule inhibitors of response regulator activation lacked assays to evaluate if targeted TCSs were effectively and specifically downregulated and thereby conferred significant biological effects (50, 53).

*A. baumannii* BfmR is an appealing antimicrobial target, because its inhibition would significantly decrease *in vivo* survival and increase sensitivity to antimicrobials. In addition, it has a lack of significant sequence similarity to any human protein, and BfmR belongs to a protein family absent from humans and other mammals. Lastly, the BfmR sequence is highly conserved across *A. baumannii* strains. However, several important questions remain to be addressed before BfmR can be classified as an established target. Thus far, no receiver domain from the OmpR/PhoB family has been demonstrated as a druggable target (i.e., activity can be modulated by a drug-like small molecule). Likewise, determining the degree of BfmR inhibition needed to impact growth/survival, an efficient BfmR activity assay for high-throughput screening BfmR activity against a small-molecule library, and an assay for determining specificity of the mechanism of action are needed for progress of BfmR as a potential new antimicrobial target. Additionally, major impediments for development of an antimicrobial effective against GNB are permeability and efflux, and a small-molecule inhibiting BfmR activity must overcome these barriers. Despite these challenges, an intriguing aspect of TCS response regulators as antimicrobial targets is the possibility of developing drugs effective against either a narrow or broad spectrum of bacteria. Additionally, the development of small-molecule tool compounds that modulate activities of response regulators will allow chemical biology dissection of bacterial signaling pathways responsive to environmental stimuli. The OmpR/PhoB family receiver domains in response regulators are widely present in bacterial pathogens and possess a degree of shared homology at the dimer interface and phosphorylation site. Therefore, it is possible that a drug inhibiting BfmR activity through its receiver domain will also inhibit the activity of additional TCS response regulators. This multitarget approach may increase durability and overcome a major concern in drug development, the development of resistance. Taken together, data presented in this report support further studies designed to develop BfmR as a drug target, including development of assays required for further target validation.

## MATERIALS AND METHODS

### Bacterial strains.

*A. baumannii* strain AB307-0294 (blood isolate; sequence type 15, clonal group 1, based on the methods of Ecker et al. [54]) has a K1 capsular serotype (55) and was isolated from a patient hospitalized at Erie County Medical Center, Buffalo, NY, in 1994 (56). The genome of AB307-0294 has been fully sequenced, and it contains 3.76 Mbp with 3,531 predicted open reading frames (57). Previous comparisons with *A. baumannii* strains in the public domain support AB307-0294 as being clinically appropriate, representative of contemporary *A. baumannii* strains, and an ideal background to identify new or unrecognized antimicrobial targets of interest (24).

AB307.70 is an isogenic BfmR-minus derivative of AB307-0294 generated by transposon mutagenesis, and it possesses a disruption in *bfmR* (nucleotides 1 to 717) due to the transposon insertion after nucleotide 592. *bfmR* putatively encodes a response regulator protein, BfmR (24). Polar effects were excluded, as described previously (24), and the complemented derivative, AB307.70/pNLAC[*bfmR*], was generated for this study to confirm that the observed phenotypic differences between AB307-0294 and AB307.70 were due to BfmR. A second BfmR-minus derivative was created by site-directed mutagenesis of the clinically relevant isolate AB908 (obtained from Walter Reed Medical Hospital) by using a one-step chromosomal gene inactivation method described elsewhere (58). In brief, a linear PCR-generated amplicon (forward primer, 5′-ATGAGCCAAGAAGAAAAGTTACCAAAGATTCTGATCGTTGAAGACGACGAGCGTTTAGCGCGATTAACTCAAGAATATTTAATCCGTAATGGTTTGGAAGTTGGTGTAGAAACCGATGGTAACCGT**AAAGCCACGTTGTGTCTCAAAATC**-3′; reverse primer, 5′-TTACAATCCATTGGTTTCTTTAACAAACAAGTAACCTTTACTACGTACAGTTTTAATACGTTTTGGATTTTCAGGATCATCGCCAATTTTTGGACGAATACGTGAAATACGTACGTCAATTGAACG**CATTATTCCCTCCAGGTATTAGAA**-3′; bold sequence portions are homologous to the kanamycin resistance gene sequence) that contained a kanamycin resistance cassette flanked by the first and last 125 bp of *bfmR* was electroporated into AB908/pAT02 (58), and recombinants were selected on Luria-Bertani (LB) plates containing kanamycin. The successful disruption of *bfmR* in AB908Δ*bfmR* was confirmed by sequence analysis, and a derivative cured of pAT02 was used for subsequent studies. AB307.30 and AB307::Tn*69* are isogenic derivatives generated by transposon mutagenesis that possess disruptions in *wzc* (a capsule transport gene) and *gna* (a capsule biosynthetic gene), respectively (33, 45). AB307.30 and AB307::Tn*69* were used in studies to exclude the presence of a second, non-K1 capsule. AB307-0294 (wt) and AB307::Tn*17* (Δ*lpsB*) (59), which possesses a disruption in a glycosyltransferase involved in lipopolysaccharide core biosynthesis, are both capsule positive and served as positive controls. Strains were maintained at −80°C in 50% LB medium (5 g of yeast extract, 10 g of tryptone, and 10 g of NaCl per liter) and 50% glycerol.

### Media.

The procedures for obtaining human ascites fluid and human serum were reviewed and approved by the Western New York Veterans Administration Institutional Review Board. Informed consent was obtained from healthy volunteers for blood collection. Blood underwent coagulation at room temperature for 15 min, followed by refrigeration at 4°C for 60 min to enable clot retraction. Serum was obtained from the blood after subsequent centrifugation at 4°C for 15 min at 3,000 × *g*. For the collection of ascites fluid, the Western New York Veterans Administration Institutional Review Board waived informed consent because the ascites fluid was collected from deidentified patients who were undergoing therapeutic paracentesis for symptoms due to abdominal distension. These individuals were not being treated with antimicrobials and were not infected with human immunodeficiency virus, hepatitis B virus, or hepatitis C virus. The ascites fluid was cultured to confirm sterility, divided into aliquots, and stored at −80°C. Each batch was obtained from a different patient and was designated by the date of removal. A single ascites fluid batch (obtained on 18 October 2012) was used for growth studies. For some experiments, human serum and ascites fluid were incubated at 56°C for 30 min to inactivate complement-mediated bactericidal activity (Δ56°C). BBL Müeller-Hinton II cation-adjusted (MH) broth consisted of 22 g of powder per liter (Becton, Dickinson). Minimal medium consisted of 200 ml of solution A [2.0 g (NH4)_2_SO_4_, 6.0 g Na_2_HPO_4_, 3.0 g KH_2_PO_4_, 3.0 g NaCl, 0.011 g Na_2_SO_4_], 800 ml of solution B (0.2 g MgCl_2_, 0.0132 g CaCl_2_ ⋅ 2H_2_O, 0.0005 g FeCl_3_ ⋅ 7H_2_O, 2.9241 g citrate [trisodium salt dehydrate]), and 3 g Casamino Acids. AB307.70 was grown in the presence of 40 µg/ml of kanamycin, and AB307.70/pNLAC[*bfmR*] was grown in the presence of 40 µg/ml of kanamycin and 200 µg/ml of carbenicillin.

### *In vitro* and *ex vivo* growth in LB medium, human ascites fluid, and human serum.

Growth experiments in LB medium, human ascites fluid, and human serum were performed as described elsewhere (56), with aliquots removed for bacterial enumeration at various times. For growth studies, 100% LB medium, 90% ascites fluid–10% 1× phosphate-buffered saline (PBS; pH 7.4), and 90% serum–10% 1× PBS were used.

### Analysis of capsule production.

AB307-0294 (wt) and AB307.70 (Δ*bfmR*) cells were grown overnight in LB medium, minimal medium, or human ascites fluid. The next day, an aliquot was removed for enumeration and bacterial cells were concentrated by centrifugation and resuspended in 50 µl/1 ml of culture lysis buffer (2% SDS, 10% glycerol in 1 M Tris [pH 6.8], to which fresh 4% β-mercaptoethanol was added). Cells in lysis buffer were boiled for 10 min. Next, the lysate was treated with 20 µl/50 µl of lysate with proteinase K (1.25 mg/ml) for 120 min at 60°C. Supernatant was generated by precipitation with 5 volumes of ice-cold 75% ethanol, which was placed at 4°C for 18 h. The precipitant was recovered by centrifugation at 8,000 × *g* for 30 min at 4°C. The supernatant was discarded and the tubes were air dried. The dried precipitates were resuspended in lysis buffer at the corresponding volume of the concordant pellet, boiled for 10 min, and treated with proteinase K in the same manner as the pellets. The cell pellet and supernatant volumes used for gel loading were normalized to a bacterial titer of 3.9 × 10^6^ CFU by dilution in lysis buffer. Capsular polysaccharide was resolved by SDS–8% polyacrylamide gel electrophoresis and subjected to Western blot analysis with MAb 13D6, which is directed against the AB307-0294 capsule (33), or were stained with alcian blue.

### Antimicrobial susceptibility testing.

*A. baumannii* strains were grown for 18 h in MH broth at 37°C. Bacteria were diluted 1:100 in the same medium, and titers were determined by serial 10-fold dilutions and enumerated on LB plates with or without appropriate antibiotics. Assays were performed in 96-well microtiter plates. Wells contained either 0 µg/ml, 0.0078 µg/ml, 0.0156 µg/ml, 0.0312 µg/ml, 0.0625 µg/ml, 0.125 µg/ml, 0.25 µg/ml, 0.5 µg/ml, 1.0 µg/ml, or 2.0 µg/ml of meropenem or 0 µg/ml, 0.0312 µg/ml, 0.0625 µg/ml, 0.125 µg/ml, 0.25 µg/ml, 0.5 µg/ml, 1.0 µg/ml, 2.0 µg/ml, 4.0 µg/ml, or 8.0 µg/ml of colistin or tigecycline in 100 µl of MH broth to which 100 µl of the same medium containing bacteria was added. Final concentrations of bacteria were 2.4 × 10^5^ CFU/ml for AB307-0294, 5.0 × 10^5^ CFU/ml for AB307.70, and 1.8 × 10^5^ CFU/ml for AB307.70/pNLAC[*bfmR*] for meropenem studies; 6.2 × 10^5^ CFU/ml for AB307-0294, 8.9 × 10^5^ CFU/ml for AB307.70, and 6.8 × 10^5^ CFU/ml for AB307.70/pNLAC[*bfmR*] for colistin studies; and 7.1 × 10^5^ CFU/ml for AB307-0294, 7.9 × 10^5^ CFU/ml for AB307.70, and 1.3 × 10^6^ CFU/ml for AB307.70/pNLAC[*bfmR*] for tigecycline studies. Control wells contained MH broth only. The plates were incubated for 18 h at 37°C. The optical density at 600 nm (OD_600_) of each well was measured in a microplate spectrophotometer (SpectraMax 190; Molecular Devices) at 26°C. Two independent experiments, each with an *n* of 6, were performed for each strain and antimicrobial concentration.

### Bioinformatic analysis of BfmR sequence and structure.

The protein sequences for BfmR orthologues were generated from respective *bfmR* gene sequences. The OMA orthologue database (September 2014 release) was searched to evaluate BfmR conservation across *A. baumannii* strains and additional *Acinetobacter* species (37). BLASTp (60), as implemented on the NCBI server (http://blast.ncbi.nlm.nih.gov), and Clustal Omega (61) were used to align sequences, and ESPript 3.0 (62) was used to display protein sequences. FTMap was used to predict locations of small-molecule binding hot spots on the exposed surface of the BfmR receiver domain, using the default set of small organic molecular probes (16 chemicals; mass range, 30.1 to 106.1 Da) (43). AutoDock Vina v. 1.1.2 and PyRx v. 0.8 were used for *in silico* screening of the BfmR receiver domain against a virtual library of drug and drug-like molecules (63, 64). The virtual molecular libraries represented small-molecule chemicals approved for clinical trials or clinical use (3,180 chemicals; mass range, 60.1 to 829.0 Da), the NCI Diversity 3 collection (1,880 chemicals; mass range, 114.1 to 697.1 Da), and the LOPAC1280 collection (1,280 chemicals; mass range, 59.1 to 836.4 Da), with molecular descriptor files obtained from the ZINC database (65).

### Recombinant protein expression and purification.

*E. coli* strain DH5α (Invitrogen) was used during construction of the *bfmR* expression vector, and strain BL21(DE3) (Novagen) was used for recombinant protein expression. PCR amplification of the portion of the *bfmR* open reading frame (NCBI accession number ACJ59134), coding for the N-terminal receiver domain (BfmR^1-130^), was acccomplished using purified AB307-0294 chromosomal DNA (RefSeq NC_011595.1) as the template and the primers forward, 5′-GCGCGACATATGATGAGCCAAGAAGAAAAGTTA-3′; and reverse, 5′-ATATATCTCGAGTTATTCAACAGTTTTATCCGTACG-3′, which included an NdeI and an XhoI cleavage site, respectively. Gel-purified PCR products were restriction enzyme digested and ligated using T4 DNA ligase (Promga) into a linearized customized pET15b-TEV (tobacco etch virus) vector containing an N-terminal His_5_ affinity tag plus a TEV protease cleavage site replacing the usual thrombin site (pET15b-TEV-*bfmR*_*rec*). The integrity of the expression cassette was verified by DNA sequencing (Roswell Park Cancer Institute Sequencing Facility).

*E. coli* BL21(DE3) cells transformed with pET15b-TEV-*bfmR*_*rec* were grown to an OD_600_ of 0.8 at 30°C in LB medium supplemented with 100 µg/ml ampicillin. Protein expression was induced with 1 mM β-d-1-thiogalactopyranoside (IPTG). The culture was incubated for an additional 5 h prior to harvesting by centrifugation. Frozen cell pellets were thawed and resuspended in lysis buffer (20 mM Tris-HCl [pH 8.0], 200 mM NaCl, 10 mM imidazole, and 1 mM β-mercaptoethanol). Cells were lysed using a Microfluidizer (Microfluidics). Filtered lysate supernatant was loaded onto a HisTrap immobilized metal ion affinity chromatography (IMAC) column (GE) and eluted with a 10 mM-to-300 mM imidazole gradient plus lysis buffer. Elution fractions containing His_5_-BfmR^1-130^ were dialyzed into lysis buffer plus 60 mM imidazole. TEV protease was added to the fusion protein at a 1:100 mass ratio, and the mixture was incubated overnight at 4°C (66). The sample was reapplied to a HisTrap IMAC column to separate the cleaved His_5_ affinity tag from BfmR^1-130^. Eluted BfmR^1-130^ was dialyzed against 20 mM Tris-HCl (pH 8.0), 200 mM NaCl, and 1 mM dithiothreitol and then was concentrated (final concentration, 11.25 mg/ml) by using Centricon-3 centrifugal concentrators (Amicon). Small aliquots were flash-frozen in liquid nitrogen and stored at −80°C. Protein purity was verified via SDS-PAGE.

### Crystallization.

Crystallization screening via microbatch under oil in 1,536-well microtiter plates was conducted using the high-throughput robotics at the Hauptman-Woodward Institute (67). Selected hits were optimized via manual sitting-drop vapor diffusion. Crystals used for diffraction data collected were obtained by equilibration at 20°C of a crystallization drop containing 2 µl of purified BfmR^1-130^ at 11.25 mg/ml plus 2 µl of reservoir solution against 1 ml of reservoir (20% [vol/vol] polyethylene glycol 400, 20% [vol/vol/] glycerol, 100 mM Tris-HCl [pH 8.5], and 150 mM NaCl).

### X-ray diffraction data collection and processing.

Crystals were harvested using a nylon loop and then subsequently flash-cooled in liquid nitrogen. No additional cryoprotectant was required due to the composition of the reservoir solution. Crystals were shipped to the Stanford Synchrotron Radiation Lightsource (SSRL), where diffraction data were collected remotely at −173°C by using a MARmosaic 325 charge-coupled-device detector on beamline 9-2. Data were collected using an X-ray wavelength of 0.97946 Å, a crystal-to-detector distance of 330 mm, a φ range of 180.4° by 0.55° increments, with 5-s exposures per frame. Data were indexed, integrated, and scaled using HKL2000 (68).

### Statistical analyses.

Continuous data were assessed for normality and are presented as means ± standard errors of the means (SEM.) *P* values of 0.05/*n* (*n* = the number of comparisons) were considered statistically significant based on the Bonferroni correction for multiple comparisons. To normalize *ex vivo* growth/survival data (Fig. 1), log_10_-transformed values were utilized, the area under each curve was calculated, and these areas were compared using two-tailed unpaired *t* tests (Prism 4 for MacIntosh; GraphPad Software, Inc.).

### Structure solution and refinement.

The receiver domain from the response regulator PhoP from *Bacillus subtilis* (PDB 1MVO) was used as the search model in molecular replacement, following removal of associated solvent and ions. The Auto-Rickshaw server (69) was used to determine the molecular replacement solution, which was automatically fed to Arp/wARP (70) to produce an initial BfmR model. Refinement was conducted using Phenix, including TLS refinement to model anisotropic displacements and noncrystallographic symmetry (NCS) restraints (71). Manual model building and analysis were performed using Coot (72). Structure analysis and validation made use of PyMol (Schrödinger, LLC), Phenix (71), PISA (73), and the validation tools present in the wwPDB deposition tool (http://deposit.wwpdb.org/deposition).

### Protein structure accession number.

The refined coordinates and scaled diffraction data have been deposited in the PDB (PDB code 5E3J).
